# Imprecise Uncertain Reasoning: A Distributional Approach

**DOI:** 10.3389/fpsyg.2018.02051

**Published:** 2018-10-26

**Authors:** Gernot D. Kleiter

**Affiliations:** Fachbereich Psychologie, Universität Salzburg, Salzburg, Austria

**Keywords:** uncertain reasoning, judgment under uncertainty, probability logic, imprecise probability, second-order distributions, coherence

## Abstract

The contribution proposes to model imprecise and uncertain reasoning by a mental probability logic that is based on probability distributions. It shows how distributions are combined with logical operators and how distributions propagate in inference rules. It discusses a series of examples like the Linda task, the suppression task, Doherty's pseudodiagnosticity task, and some of the deductive reasoning tasks of Rips. It demonstrates how to update distributions by soft evidence and how to represent correlated risks. The probabilities inferred from different logical inference forms may be so similar that it will be impossible to distinguish them empirically in a psychological study. Second-order distributions allow to obtain the probability distribution of being coherent. The maximum probability of being coherent is a second-order criterion of rationality. Technically the contribution relies on beta distributions, copulas, vines, and stochastic simulation.

## 1. Introduction

### 1.1. Logic, probability, and statistics in models of human reasoning

Fifty years ago Peterson and Beach (1967) wrote a paper with the title “Man as an intuitive statistician.” In the time before the heuristics-and-biases paradigm human judgments and decisions were seen on the background of Baysian statistics. In the same time human reasoning was exclusively seen on the background of classical logic. The Wason task became a prototypical experimental paradigm. One might have written a paper with the title “The human reasoner as an intuitive logician.” This changed from the middle of the 1990s when probability entered the scene of human reasoning research. In 1993 *Cognition* published a special issue on the interaction between reasoning and decision making (Johnson-Laird and Shafir, 1993) with contributions, among others, by Johnson-Laird, Tversky, or Evans. Shortly afterwards Oaksford and Chater (1995) proposed to model the Wason task in terms of probabilistic information seeking. In the same year Over investigated the suppression task in terms of probabilities (Stevenson and Over, 1995). Before that time reasoning research was exclusively done on the background of logical benchmarks, while judgment under uncertainty, however, was investigated on the background of probabilistic and decision theoretic benchmarks. Reasoning investigated the human understanding of material implications (like in the Wason task), propositional inference rules (like the modus ponens), inferences with quantifiers (like syllogisms), and the validity of inference forms. The modus ponens, for example, was not cast into a probabilistic format (except by George Boole more than 100 years earlier). The judgment under uncertainty community investigated updating probabilities via Bayes' theorem, calibration, and later on the heuristics and biases. Logicians had already started probability logic and default reasoning in the 1960s (Adams, 1965, 1966; Suppes, 1966)^1^.

In judgment under uncertainty logical rules like the modus ponens or the modus tollens were not investigated. Inference forms of classical logic could not directly be cast into a probabilistic format. First, there was the problem of conditionals. In classical logic a conditional is a material implication. In probability logic the conditional is a conditional event to which a conditional probability may be assigned. Conditional events, however, are outside of classical logic. Second, probabilistic inference is not “truth-functional” in a way that is analog to classical logic. In classical logic the truth values of the premises determine the truth-value of the conclusion. If *A* is true and *A* → *B* is true, then *B* is true. In probability theory the probabilities of the premises of a modus ponens do not exactly determine the probability of its conclusion; the premises only constrain the probability of the conclusion by lower and upper probabilities. If *P*(*A*) = *x* and *P*(*B*|*A*) = *y*, then *xy* ≤ *P*(*B*) ≤ 1−*x* + *xy*. Research on mental probability logic and the new (probabilistic) paradigm after the middle of the 1990s might have been published under the title “The human reasoner as an intuitive probabilist.” At conferences one could follow discussions on questions like “should binary truth values be basic ingredients in models on human reasoning?”

No doubt, the adoption of probability extended and enriched the research on human reasoning. However, probability combined with some logic is still insufficient to model reasoning and decision making in a complex and uncertain environment. The reasoner as an “intuitive statistician” is missing. The intuitive statistician is required when it comes to learning, to prediction, and to decision making. A typical problem that cannot be handled in elementary probability logic but than can conveniently be handled in statistics is the *distributional precision*. By distributional precision I mean the spread-out and dispersion of a continuous distribution around a favorite value. Mental probability logic assumes precise point probabilities or probability intervals where the lower and upper bounds are again precise. Representing imprecise uncertainties by distributions opens the door to invoke an interface to frequencies observed in the outside world. We will borrow the tool of beta distributions from Bayesian statistics. Their use in psychological modeling has the advantage of providing the possibility to update beliefs in the light of new evidence and observed frequencies. “…the true power of a probabilistic representation is its ability not only to deal with *imprecise* probability assessments, but to welcome them as providing a natural basis for the system to improve with experience” (Spiegelhalter et al., 1990, p. 285). In Pfeifer and Kleiter (2006a) we used mixtures of beta distributions to model inferences with imprecise probabilities.

The present paper proposes first steps toward a mental probability logic based on distributions. It employs second-order probability distributions and some more recent concepts of modeling probabilistic dependence by copulas and vines. Human reasoners and decision makers should be seen as a combination of intuitive logicians, of intuitive probabilists, and of intuitive statisticians. All three levels should be addressed in the basic research questions, in the experimental paradigms, and in the normative models.

Imprecision may be expressed by various distributions. One option, for example, is the family of log-normal distributions. We made a different choice and decided for beta distributions, a family of distributions that seems to be simpler and more flexible than the log-normal. So let us, at the outset, give a short characterization of the beta family.

### 1.2. Beta distribution

Throughout the contribution we will express imprecise probabilities by beta distributions. Beta distributions build a rich and flexible family of probability density functions (Johnson and Kotz, 1970; Gupta and Nadarajah, 2004). An uncertain quantity *X* is (standard) beta distributed in the interval [0, 1] with shape parameters *α* and *β* if

(1) $$\begin{array}{r} p\left(x\right)=\frac{\text{Γ}\left(\alpha +\beta \right)}{\text{Γ}\left(\alpha \right)\text{Γ}\left(\beta \right)}x^{\alpha -1}{\left(1-x\right)}^{\beta -1},\;0\leq x\leq 1. \end{array}$$

For integer values the ratio of gamma functions simplifies to (*α* + *β* − 1)!/[(*α* − 1)!(*β* − 1)!]. We write for short *X* ~ Be(*α*, *β*). The mean and the variance of the distribution are

(2) $$\begin{array}{r} \text{E}\left(X\right)=\frac{\alpha }{\alpha +\beta }\text{ and }\text{Var}\left(X\right)=\frac{\alpha \beta }{{\left(\alpha +\beta \right)}^{2}\left(\alpha +\beta +1\right)}. \end{array}$$

**Figure 1:**
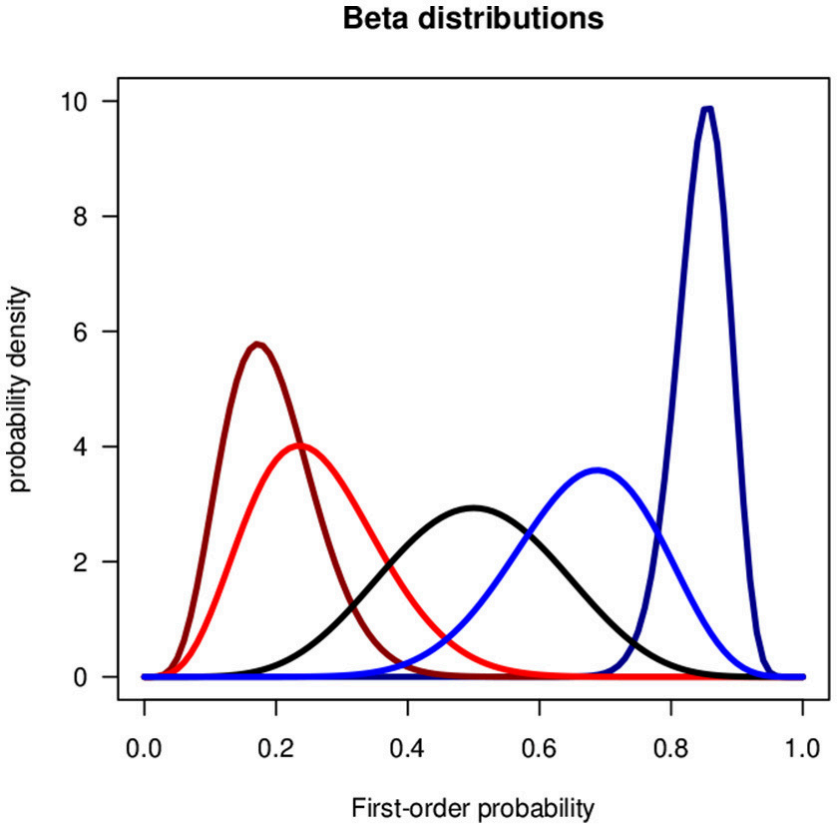
Beta distributions for the verbal phrases of the Lichtenstein and Newman data in Table 1. From **(left)** to **(right)**: Very unlikely, unlikely, about as likely as not, likely, very likely.

In the present context the random variable *X* is a first-order probability and *p*(*X*) is a second-order probability density function. In Bayesian statistics the shape parameters *α* and *β* are related to the frequencies of success and failure. *α* and *β* may be interpreted as weights of evidence, the pros and contras for a binary event, or as real or hypothetical samples sizes. *Be*(1, 1) is the uniform distribution. If *α* > 1 and *β* > 1 the distributions is uni-modal, if either *α* < 1 or *β* < 1 it is J-shaped, and if *α* < 1 and *β* < 1 it is U-shaped. Figure 1 shows uni-modal examples.

While beta distributions do not arise exclusively in Bayesian statistics, Bayesian statistics is the field in which they are most prominent. For the assessment of subjective probability distributions Staël von Holstein proposed to fit beta distributions to quantiles 1970 and before (Staël von Holstein, 1970; Kleiter, 1981). Thomas Bayes was actually the pioneer of beta distributions in his investigation of an uncertain probability (Bayes, 1958).

The next section gives a motivating example of the application of beta distributions. Imprecision is contained in the verbal uncertainty phrases we use in everyday conversation and beta distributions may be used to represent the imprecision in a mathematical form.

### 1.3. Verbal uncertainty phrases

Practically all human probability judgments are imprecise. Take the following phrases in everyday communication: “very probably,” “pretty sure,” “highly unlikely,” and so on. Verbal phrases are not only used to express degrees of belief in everyday conversation, they are also used to communicate expert knowledge, for example in geopolitical forecasting (Friedman et al., 2018) or in climate research. The Climate Science Special Report of the United States Government's (Wuebbles et al., 2017) reports a list of Key Findings. In the Climate Report each Key Finding is weighted by a verbal phrase for its likelihood. The “semantics” given to each of the phrases are shown in Table 1.

“The frequency and intensity of extreme heat and heavy precipitation events are increasing in most continental regions of the world (*very high confidence*). These trends are consistent with expected physical responses to a warming climate. Climate model studies are also consistent with these trends, although models tend to underestimate the observed trends, especially for the increase in extreme precipitation events (*very high confidence* for temperature, *high confidence* for extreme precipitation). The frequency and intensity of extreme high temperature events are virtually certain to increase in the future as global temperature increases (*high confidence*). Extreme precipitation events will very likely continue to increase in frequency and intensity throughout most of the world (*high confidence*). Observed and projected trends for some other types of extreme events, such as floods, droughts, and severe storms, have more variable regional characteristics” Wuebbles et al. (2017, p. 35).

**Table 1:** Verbal uncertainty phrases (Row A) and their numerical interpretation (Row B) as used in the US Government's climate report [Wuebbles et al. (2017, p. 35)].

**A**	**Exceptionally**	**Extremely**	**Very**	**Unlikely**	****About as ****	**Likely**	**Very**	**Extremely**	**Virtually**
	**unlikely**	**unlikely**	**unlikely**		****likely as not****		**likely**	**likely**	**certain**
B	0–1%	0–5%	0–10%	0–33%	33–66%	66–100%	90–100%	95–100%	99–100%
C			10% (7%)	16% (10%)	50% (13%)	75% (11%)	90% (4%)		
D			Be(6, 25)	Be(5,14)	Be(7,7)	Be(12,6)	Be(66, 12)		

*Row C, Medians and standard deviations (in parentheses) of the interpretation of the same verbal phrases as in Row A by 180 persons in the study of Lichtenstein and Newman (1967). Row D, Shape parameters of the fitted beta distributions shown in Figure 1*.

One of the first empirical studies on the interpretation of verbal uncertainty phrases in terms of numerical probabilities was performed by Lichtenstein and Newman (1967). Table 1 shows the medians and standard deviations of the distributions of the responses of 180 persons. We represent the verbal uncertainty phrases by beta distributions. Figure 1 shows the beta distributions fitted to the medians and standard deviations of the data.

There are two different directions in which imprecise uncertainty can be modeled, by down-shifting or by up-shifting. Down-shifting relaxes the precision of the description and works with qualitative or comparative probabilities. Baratgin et al. (2013), for example, investigated human reasoning in terms of qualitative probabilities. Up-shifting refines the level of the description on a meta-level. Describing imprecise uncertainty by distributions, as proposed in the present contribution, is an example of up-shifting.

The elementary theorems of probability theory propagate precise probabilities of the premises to precise probabilities of the conclusions. If, for example, *A* and *B* are two probabilistically independent events and *P*(*A*) = *x* and *P*(*B*) = *y*, then *P*(*A*∧*B*) = *z* = *x*·*y*. If probabilities are introduced in elementary *logical* operators or theorems, however, precise probabilities of the premises propagate to *imprecise* probabilities of the conclusions. If the two events *A* and *B* are not probablistically independent then the probability of *A*∧*B* is an interval probability, *P*(*A*∧*B*) = *z*∈[max{0, *x*+*y*−1}, min{*x, y*}].

The theory of imprecise probabilities (Walley, 1991; Augustin et al., 2014) expresses imprecision by lower and upper probabilities, i.e., by *interval probabilities*. For psychological modeling, however, interval probabilities have several disadvantages. The iteration of conditional interval probabilities leads to theoretically complex solutions (Gilio and Sanfilippo, 2013). Moreover, empirically checking the endorsement of inferences may become too permissive because the responses of the participants may fall into very wide intervals. Another, more principal and theoretical difficulty poses the question how to base decisions on probability intervals. This problem was especially raised by Smets (1990) (for a review see Cuzzolin, 2012). Smets distinguished *credal* and *pignistic* degrees of belief, the first one for contemplation and the second one for action. We will tackle the question below and propose a new criterion, the maximum probability of being coherent. But let us first turn to the question of how to incorporate and propagate distributions in the framework of basic logical operators.

## 2. Propagating imprecision in logical inferences forms

### 2.1. Elementary logical operators

If our knowledge about the probability of an event *A* is represented by the beta distribution *P*(*A*) ~ *Be*(*α*, *β*), then our knowledge about its negation ¬*A* should be expressed by *P*(¬*A*) ~ *Be*(*β*, *α*). The parameters *α* and *β* just switch positions.

In many investigations (see for example Kleiter et al., 2002) it was observed that probability assessments of *A* and ¬*A* do not add up to 1. If the participants of an experiment assess the probability of *A* and after a while give an assessment of ¬*A* then usually *P*(*A*)+*P*(*B*) ≠ 1.0. Probability judgments of “Is New York north of Rome?” and “Is Rome north of New York?” may easily lead to superadditivity, *P*_1_ + *P*_2_ > 1. Deviations from 1.0 may be systematic or random. Poor experimental conditions contribute to low reliability and next-best judgments. Erev et al. (1994) have shown that low reliability of probability judgments may lead to overconfidence and hyper-precision.

Let us next consider logical conjunction. For precise probabilities of the premises we have

(3) $$\begin{array}{l} \text{If }P\left(A\right)=x\;\text{and }P\left(B\right)=y,\\ \text{then }P\left(A\wedge B\right)=z\in \left[\max\left\{0,x+y-1\right\},\min\left\{x,y\right\}\right]. \end{array}$$

**Figure 2:**
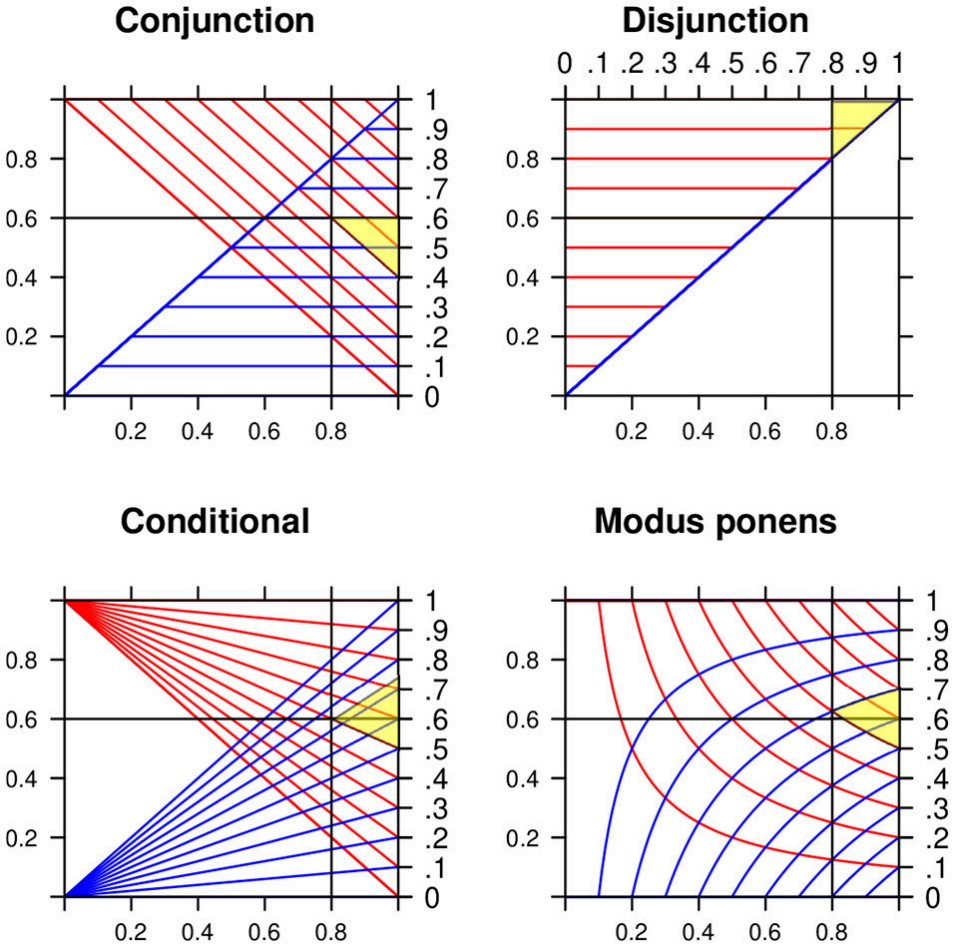
Lower and upper probabilities for the conjunction, the disjunction, the conditional with *P*(*A*) = *X* and *P*(*B*) = *Y*, and the modus ponens with *P*(*A*) = *X* and *P*(*B*|*A*) = *Y*. Numerical example for *x* = 0.8 and *y* = 0.6 (for the MODUS PONENS slightly above 0.6). The yellow shadowed areas indicate the projections to the intervals [0.4, 0.6], [0.8, 1], [0.5, 0.7], and [0.5, 0.7].

The lower and the upper bounds are known as the two Fréchet-Hoeffding copulas (Nelsen, 2006). Any probability assessment *z* in the interval is *coherent*. A probability assessment is coherent if it does not lead to a Dutch book (losing for sure). The top left panel in Figure 2 shows lines for equal lower (upper) probabilities as functions of the marginals *P*(*A*) and *P*(*B*). At (0.8, 0.6) the probabilities “project” to the interval [0.4, 0.6].

Next we replace the precise probabilities *x* and *y* by the two random variables *X* and *Y*, where *X* ~ *Be*(*α*_1_, *β*_1_) and *Y* ~ *Be*(*α*_2_, *β*_2_). Moreover, we specify the kind and the degree of dependence between *X* and *Y* by a copula *C*(*x, y*). To keep the contribution as simple as possible we will use Gaussian copulas, that is, Pearson's correlations. The coefficients will be denoted by *ρ*. There are many other copulas (Nelsen, 2006). The two marginal distributions of *X* and *Y*, together with the copula *C*(*x, y*), determine the joint distribution with the densities *p*(*x, y*) on the unit square [0, 1]^2^. The bivariate Gaussian copula with the correlation coefficient *ρ* is given by

(4) $$\begin{array}{l} C(u,v)=N_{\rho }(\Phi^{-1}(u),\Phi^{-1}(v))\\ \;=\frac{1}{2\pi \sqrt{1-\rho^{2}}}\int_{-\infty }^{\Phi^{-1}(u)}\text{​}\text{​}\int_{-\infty }^{\Phi^{-1}(v)}\text{​}\text{​}exp\left[-\frac{1}{2}\left(\frac{s^{2}-2\rho st+t^{2}}{1-\rho^{2}}\right)\right]dsdt \end{array}$$

with $s=\frac{u-\mu_{u}}{\sigma_{u}}$ and $t=\frac{v-\mu_{v}}{\sigma_{v}}$ and Φ^−1^(*u*) and Φ^−1^(*u*) denote the inverse of the univariate standard normal distribution function.

The unit square is analog to the 2 × 2 truth table in classical logic. While a truth table has only the two values 0 and 1 on its margins, the unit square has the real numbers between 0 and 1 along its two margins. In logic an operator maps the entries from the 2 × 2 table into {0, 1}. In the distributional approach an operator maps the densities on the unit-square to densities on the [0, 1]-interval. The two place operators require two mappings, one for the lower bound and one for the upper bound.

Each fixed value of the lower probability in (3) determines a contour line in the joint distribution on the unit square. Collecting the densities along such a contour line gives the probability density for a fixed value of the lower probability. And the same holds for the upper probability. So we get two distributions, one for the lower and one for the upper probabilities. Technically in most cases these steps cannot be performed analytically in closed form. We use a stochastic simulation method implemented in the VineCopula package (Mai and Scherer, 2012; Schepsmeier et al., 2018) of the statistical software R (R Development Core Team, 2016). The R code of program for the analysis of the four inference forms discussed below is contained in the Supplementary Material.

**Figure 3:**
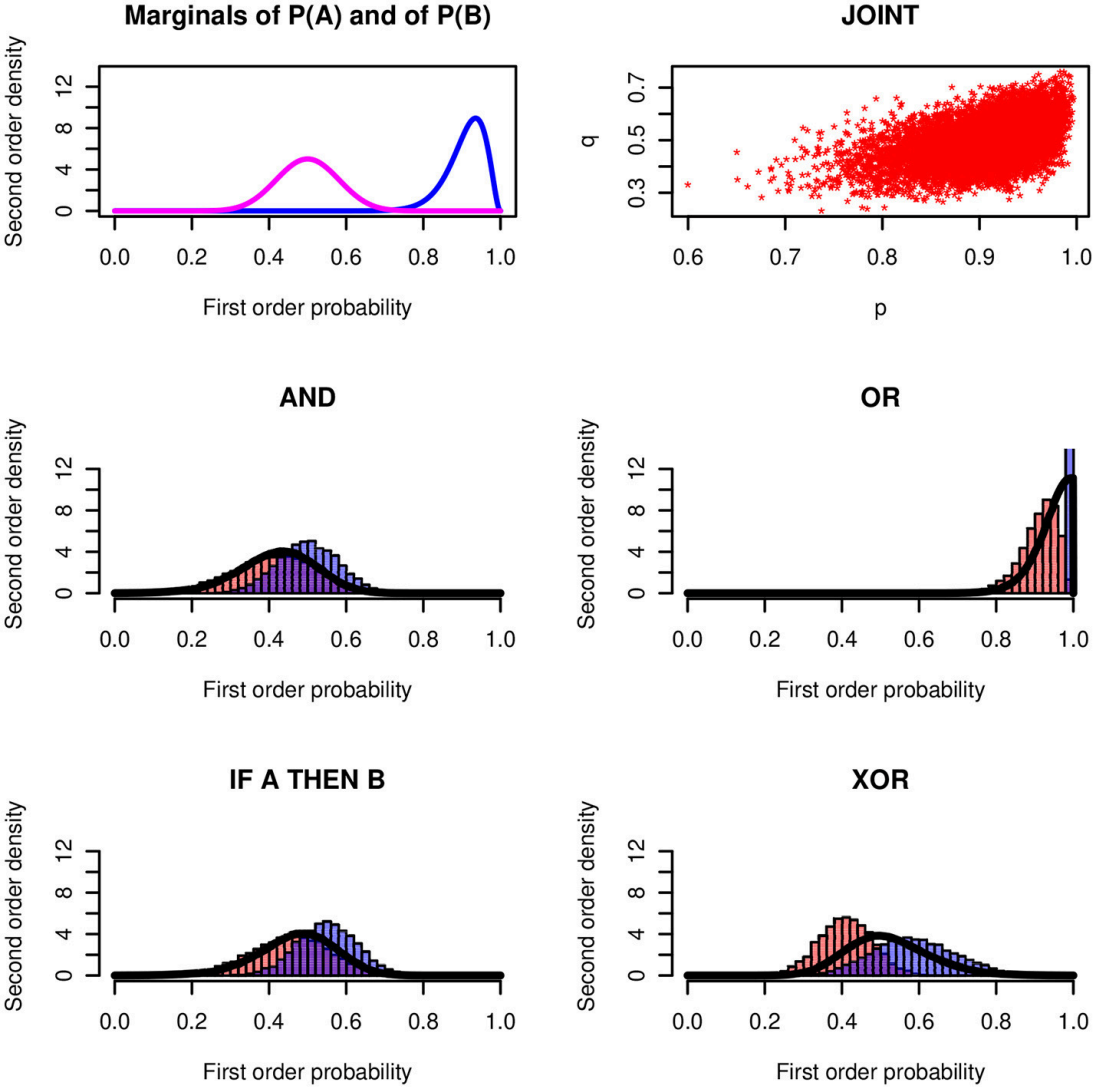
Basic logical operators. **(Top row, Left)** Premises *P*(*A*) ~ *Be*(30, 3) and *P*(*B*) ~ *Be*(20, 20). **(Right)** Scatter diagram of the joint distribution with Gaussian copula *ρ* = 0.5. **(Middle and Bottom row)** Histograms of the lower and upper probabilities for and, or, if-then, and xor operators together with the bold lines showing the probability of being coherent. The upper probability of the disjunction degenerates at 1.

We applied the stochastic simulation method to the conjunction, the disjunction, to the conditional event interpretation of the conditional (if *A*, then *B* means *B*|*A*) and to the exclusive disjunction. Figure 3 shows a numerical example for each one of the four operators. The distributions of the probabilities of *X* ~ *Be*(30, 3) and of *Y* ~ (20, 20) are plotted in the left panel of the top row. The two first-order probabilities are correlated with the Gaussian copula *ρ* = 0.5. The scatter diagram shows the simulation of 10,000 points of the joint distribution on the unit square.

The histograms in the four panels show the relative frequencies of the lower and upper bounds resulting from the simulations. The continuous distributions approximate the probability density of being coherent. This is a meta-criterion. It corresponds to the probability that the value of a first-order probability assessment falls into the coherent interval between the two Fréchet-Hoeffding bounds. The concept will be explained below.

To consider correlations between probabilities may require a short comment. Probabilities may provide information about other probabilities. Take as an example co-morbidity in age-related diseases. Diabetes, Parkinson's and Alzheimer's disease often come together (Bellantuono, 2018). If we are 90% sure that an elderly person gets diabetes we infer that the probability that the person gets Parkinson's disease rises to a value above average. The probabilities of having the two diseases are correlated. Risks may be correlated. Assume the father of a male person suffers from prostate cancer. Knowing that the probability of having inherited some of the critical gens is high, increases the risk that the person will get prostate cancer.

Figure 3 shows a stunning result: The conjunction and the conditional (with conditional event interpretation) lead to nearly the same results^2^. It will not be possible to distinguish the two operators empirically in a psychological study. For a speaker who expresses imprecise uncertainties the *if-then* and the *and* have practically the same “meaning.” This throws a new light on the conjunctive interpretation of conditionals. In Fugard et al. (2011) and Kleiter et al. (2018) we observed that about twenty percent of the participants give conjunctive interpretations of the conditional. We also observed a higher frequency of conjunctive interpretations in female participants. In real life communication, where most content is uncertain and the uncertainty is imprecise, this may not make a practical difference. We will come back to this question below after we will have introduced the distribution of being coherent.

**Figure 4:**
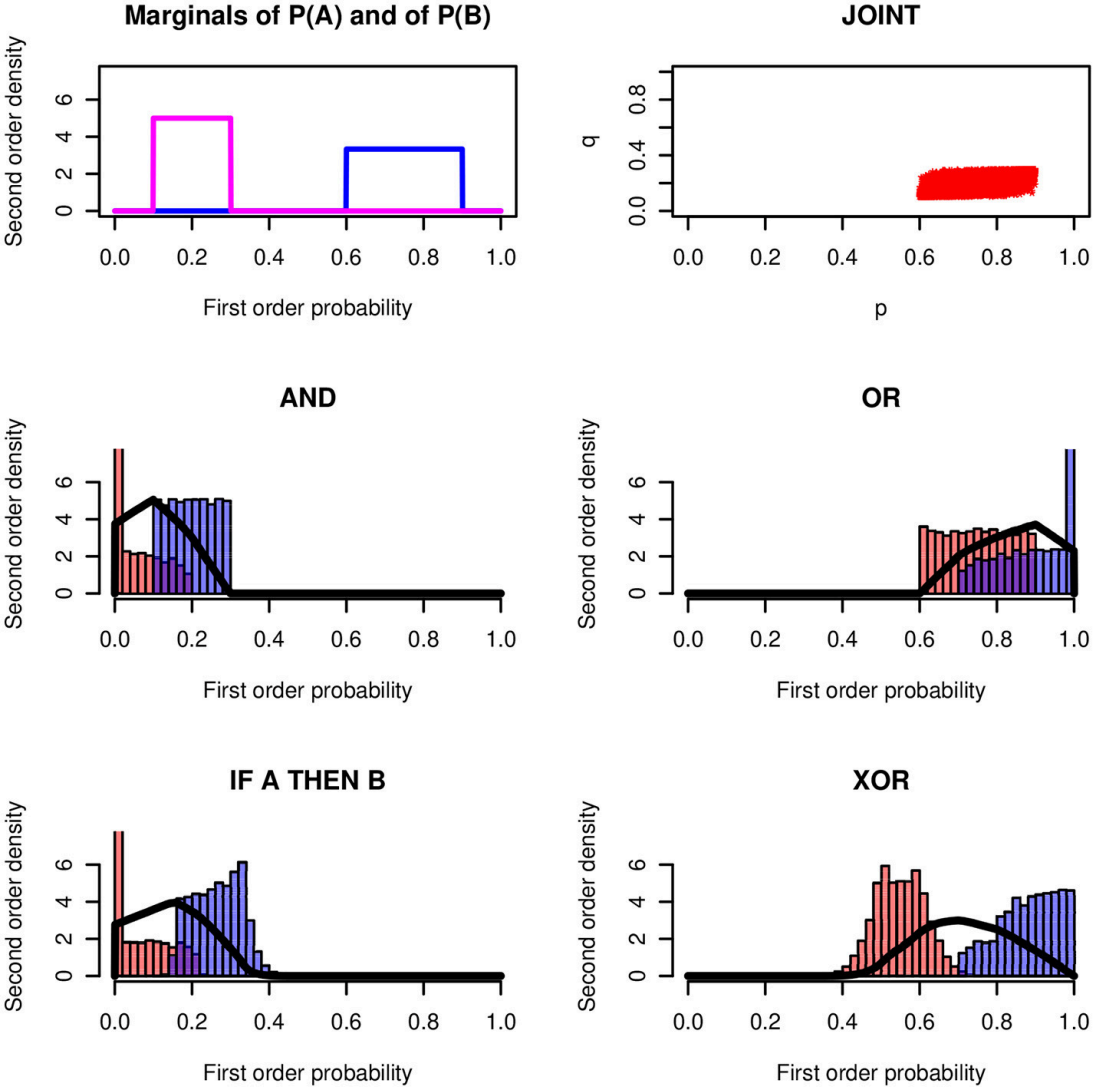
Logical operators applied to rectangular distributions *Re*(0.60, 0.90) and *Re*(0.10, 0.30) and *ρ* = 0.7. The modes of the four probability-of-coherence distributions are 0.101, 0.901, 0.157, and 0.701, respectively.

Figure 4 shows the results for an example with rectangular distributions. It assumes rectangular distributions of *X* and *Y* on the intervals *Re*[*l*_1_, *u*_1_] and *Re*[*l*_2_, *u*_2_]. Again, the conjunction and the conditional are so similar that they cannot be distinguished empirically.

Before we proceed with a discussion of the conjunction fallacy we introduce the concept of the probability of being coherent. The conjunction fallacy focuses on errors. The probability of being coherent focuses on coherent probability assessments.

### 2.2. The probability of being coherent

Probabilistic inferences that mimic logical inferences lead from a set of precise coherent probabilities of the premises to coherent interval probabilities of the conclusion. Coherence means to not allow a Dutch book, i.e., a bet where you lose for sure^3^. Denote the inferred interval by [*w, m*]. All values between *w* and *m* are coherent.

In the present approach *w* and *m* are realizations of random variables. The probability for an assessment *z* to be coherent is equal to the probability that *z* is greater than *w* and less than *m*, i.e., *p*(*z*∈[*w, m*]). The distribution cannot be obtained in closed form. Numerical results are determined by stochastic simulation. Consider for example the conjunction of *A* and *B* with *P*(*A*) = *X* ~ *Be*(*α*_1_, *β*_1_), *P*(*B*) = *Y* ~ *Be*(*α*_2_, *β*_2_), and the copula *C*(*x, y*). We perform the following steps:

1. Discretize the real numbers between 0 and 1 into *n* steps; we rescale the [0, 1] interval by [0, 1, …, 1000].
2. Initialize an array *f*[0], *f*[1], …, *f*[*n*] of length *n*+1 with all values equal to 0. The array will collect frequency counts.
3. Sample two random probabilities *x* and *y* from the two beta distributions of *A* and *B*; for doing this use the copula *C*(*x, y*). Independence is a special case.
4. Determine the lower and upper bounds *w* = max{0, *x*+*y*−1} and *m* = min{*x, y*}.
5. Add 1 to the frequency count of each discretized value between *w* and *m*, *f*[*i*] = *f*[*i*]+1, *i* = 1000·*w*, …, 1000·*m*.
6. Repeat the steps 3 to 5 *N* times. *N* may, for example, be 50,000.
7. Divide the frequency counts of the discretized values by *N*. The result approximates the distribution of the probability of being coherent.

We implemented these steps in R (R Development Core Team, 2016) using the package VineCopula (Schepsmeier et al., 2018). The package offers a multitude of different copulas that may be used to specify the kind and the strength of dependencies (see also Mai and Scherer, 2012).

It is rational to require that a precise probability assessment in a probabilistically imprecise world maximizes the probability of being coherent. The second-order probabilities do not lose the Dutch book criterion as claimed by Smets and Kruse (1997, p. 243). If there is a set of bets, it is reasonable to prefer that one that maximizes the probability to avoid losses. *The hierarchical construction of first- and second-order probabilities goes hand in hand with a multi-level rationality criterion*.

Smets (1990) distinguished two levels of uncertainty representation: The *credal* level—beliefs are entertained—and the *pignistic* level—beliefs are used to act. Interval probabilities are typical of the credal level. They may be entertained in the cognitive representation of uncertainty. Practical decisions, however, require the selection of precise point values that maximize, e.g., expected utility. Smets' pignistic probabilities are different from the maximum probability of being coherent. We note that point probabilities are not always required for decision making. In decision theory, economics, and risk management *distributions* and not only exact probabilities are compared. The criterion of stochastic dominance (Sriboonchitta et al., 2010) may, for example, be applied to two distributions of being coherent.

The discriminatory sensitivity of the logical connectives may be studied by measuring the distance between two distributions of being coherent. A well known measure for the distance between two distributions is the Kullback-Leibler distance. Because of the stochastic simulation the distributions of the probability of being coherent are discrete, in our case having *N* = 1, 000 increments. The Kullback-Leibler distance between two probability distribution *P* and *Q* is given by

(5) $$\begin{array}{ll} D\left(P;Q\right)= & \overset{N}{\underset{i=1}{\sum }}P\left(x_{i}\right)\log\frac{P\left(x_{i}\right)}{Q\left(x_{i}\right)},\\ & \text{where}x_{1}=1/N,x_{2}=2/N,\ldots ,x_{N}=1. \end{array}$$

Numerical probabilities equal to zero were set equal to 0.0001. Table 2 shows the distances between ten pairs of distributions, three kinds of beta distributions, and the two correlation coefficients *ρ* = 0.5 and *ρ* = −0.5.

**Table 2:** Logical operators: Kullback-Leibler distances between the second order distributions of the probability of being coherent and the uniform distribution (UFD) and between the distributions of the conjunction (AND), the disjunction (OR), the conditional (IF) and the exclusive disjunctions (XOR).

**P(A)**	**P(B)**	**ρ**	**AND**	**OR**	**IF**	**XOR**	**OR**	**IF**	**XOR**	**IF**	**XOR**	**XOR**
			**UFD**	**UFD**	**UFD**	**UFD**	**AND**	**AND**	**AND**	**OR**	**OR**	**IF**
Be(30,3)	Be(20,20)	0.5	7.80	8.78	7.80	7.74	11,06	0.14	0.63	9.94	9.30	0.21
Be(30,3)	Be(20,20)	−0.5	8.77	7.80	7.80	7.74	11,06	0.47	0.19	9.40	9.77	0.21
Be(100,10)	Be(20,2)	0.5	8.22	9.10	8.45	8.17	6.78	3.16	10.43	0.716	10.47	10.46
Be(100,10)	Be(20,2)	−0.5	8.55	9.34	8.54	8.33	8.86	4.80	10.63	1.35	10.63	10.63
Be(20,100)	Be(5,20)	0.5	8.55	7.88	6.91	7.67	6.41	6.18	3.25	3.13	1.46	0.68
Be(20,100)	Be(5,20)	−0.5	8.66	8,12	6.92	7.77	8.73	6.51	4.82	4.16	1.98	0.74

*ρ denotes the value of the Gaussian copula*.

The left side of Table 2 contains distances from the uniform distribution (UFD). These distances are all high and relative insensitive to the kind of the distributions of *P*(*A*) and *P*(*B*) and the correlation coefficients *ρ*. The greatest distances are between or and UFD and between and and UFD.

On the right side of Table 2 small distance indicate that the probabilistic semantics of the two operators is similar. The smallest value of *D*(*P*; *Q*) = 0.14 is obtained for the distance between if and and for *P*(*A*) ~ *Be*(30, 3) and *P*(*B*) ~ (20, 20), that is, for one distribution with a high mean of 0.91 and one distribution with a mean of 0.5. This may be related to the empirical finding that about twenty percent of the interpretations of if-then sentences are conjunction interpretations (Fugard et al., 2011; Kleiter et al., 2018).

The conclusion that may be drawn from this analysis is: *The difference or the similarity of the probabilistic meaning of two logical operators depends on the high, middle, or low probabilities of the events and on the copula between the two*. This makes the empirical investigation of the semantics of the logical operators in reasoning and everyday language more difficult than often assumed. This holds, for example, for our own experiments where we used truth-table tasks in which relative frequencies were selected that may discriminate conjunctions, disjunctions, conditionals etc. This is only possible if the frequencies presented to the participants in the truth tables are close to being equally distributed and not rather high or low.

We next turn to the conjunction fallacy, one of the best known fallacies in the heuristics and biases paradigm. We will see that imprecision is a factor that may explain the fallacy at least to some degree.

### 2.3. Conjunction fallacy

In the same way as we asked for the probability of being coherent, we may ask for the probability of being incoherent. A prototypical example for incoherent probability judgments is the Linda task (Tversky and Kahneman, 1983):

Linda is 31 years old, single, outspoken and very bright. She majored in philosophy. As a student, she was especially concerned with issues of discrimination and social justice, and also participated in anti-nuclear demonstrations. Rank order the probabilities for

- Linda is a bank teller.
- Linda is active in the feminist movement.
- Linda is a bank teller and is active in the feminist movement.

Many people think the conjunction is more probable than one or even both its conjuncts. They are victims of the conjunction fallacy.

Like many other tasks in the literature on fallacies and biases, the Linda task is an example for highly imprecise probabilities. Denote “Linda is a bank teller” by *A*, “Linda is a feminist” by *B* and assume *P*(*A*) = *X* ~ *Be*(*α*_1_, *β*_1_), *P*(*B*) = *Y* ~ *Be*(*α*_2_, *β*_2_), and a Gaussian copula with *ρ* = 0.7.

You create two vague ideas of the probabilities of *A* and *B*, modeled here by two beta distributions. Next you think about reasonable values for the probabilities of the conjunction, modeled here by the distribution of the probability of being coherent. In the terminology of Smets the three distributions belong to the *credal* level. The beliefs are just “entertained” and their imprecision is part of their representation. When it is time for judgment one value *x* is sampled from the distribution for *A* and one value *y* from the distribution for *B*. Now if you really think hard you *infer* the third value *z* on the basis of *x* and *y* and the inferred value may be coherent. If you are lazy you sample a third time, now a value *z* from the distribution for being coherent. You come up with a judgment *z* that is *decoupled* from *x* and *y*. If you think hard your judgment of *z* is coupled to the precise values *x* and *y*, with less strain it is sampled from a distribution. In this case *z* may easily exceed the upper bound of the conjunction probability, i.e., the minimum of *x* and *y* and the result is a conjunction error. The probability of this one-sided incoherence corresponds to the probability that *z* is in the interval between the upper bound *m* and 1, *P*(*z*∈[*m*, 1]).

Applying simulation methods again gives a surprising result. If my probability assessment of “Linda is a bank teller” is close to 0.5 or if my assessment of “Linda is active in the feminist movement” is close to 0.5, the probability of a conjunction error may be as high as 50%. *Imprecise probabilities may induce a high percentage of conjunction errors*. If the location of the central tendency of one of the marginals is close to 0.5, then the probability of a conjunction error is close to 0.5. The probability decreases when both means move away from 0.5. The size of the correlation (or the copula parameter) does nearly not matter. Table 3 gives a few numerical examples.

**Table 3:** Probability of a conjunction error.

****Beta distribution****	****Be(1,1)****	****Be(2,2)****	****Be(4,2)****	****Be(8,2)****	****Be(16,2) ****
Probability of a conjunction error	0.50	0.50	0.33	0.22	0.15

*The beta distribution of one conjunct is held constant at Be(30, 5); Gaussian copula *ρ* = 0.5*.

We next turn to uncertain conditionals, the salt in the soup of probability logic. The interpretation of conditionals by humans was and is an especially important topic in human reasoning research. Imprecise conditionals were studied in terms of lower and upper probabilities. In the next section we will turn to distributional imprecision.

### 2.4. Conditional

Modeling conditioning with imprecise probabilities is an intricate problem. This is seen from the many different proposals made in many-valued logic, in work on lower probabilities and the Dempster-Shafer belief functions, or in work on possibilistic and fuzzy approaches. In the coherence approach inferences where the *conclusion* is a conditional require special methods. The extension of the Fundamental Theorem of de Finetti to conditional probabilities is due to Lad (1996). He also explains how numerical results are found by linear in-equalities and fractional programming (Lad, 1996).

The psychological literature reports many experiments on the interpretation of uncertain conditionals.The *truth table method* is used to distinguish between the material implication of classical logic and the conditional event interpretation. Especially the “new probabilistic paradigm” (Over, 2009; Elqayam, 2017) in reasoning research has used this task. The task is based on the truth values of the antecedent and the consequent. I, the experimenter, show you, the participant, the four combinations of the binary truth values of *A* and of *B* together with their associated probabilities. You tell me the probability you assign to “If *A* then *B*.” I infer on which truth values you were attending and this allows me to reconstruct your logical interpretation of the conditional.

Given *P*(*A*) = *x* and *P*(*B*) = *y* the probability of *P*(*B*|*A*) = *z* is in the interval

(6) $$\begin{array}{r} z\in \left[\max\left\{0,\frac{x+y-1}{x}\right\},\min\left\{1,\frac{y}{x}\right\}\right],\;x>0. \end{array}$$

The Figures 3, 4 show examples for the distribution of *P*(*B*|*A*), the probability of a conditional. We have already pointed out that the results for the conjunction and the conditional can be very similar.

For the material implication (denoted by *A* → *B*) this is different. Given *P*(*A*) = *x* and *P*(*B*) = *y* the probability of *P*(*A* → *B*) = *z* is in the interval

(7) $$\begin{array}{r} z\in \left[1-\min\left\{y,1-x\right\},\min\left\{1-y+x,1\right\}\right]. \end{array}$$

The lower and upper probabilities are equivalent to those of the disjunction ¬*A* ∨ *B*. If the probability of the antecedent *P*(*A*) is high then the distribution of the lower and upper probabilities and the probability of being coherent are very similar to the disjunction *A* ∨ *B*. With increasing *P*(*A*) the distributions of ¬*A* ∨ *B* and *A* ∨ *B* get more and more indistinguishable. In an imprecise probabilistic environment the question “material implication or disjunction?” does not matter. The question “conditional event or material implication?”, however, makes a big difference: The conditional event interpretation leads to much lower probabilities than the material implication. This is a highly relevant aspect for the interpretation of *if-then* sentences in the context of risk assessment.

The interpretation of conditionals leads us to the next section, to logical inference rules. Psychologists have often investigated the modus ponens along with the modus tollens and two logically non-valid argument forms.

### 2.5. The MP-quartet

Four inference rules were often investigated in the psychology of human reasoning: The quartet of the modus ponens, the modus tollens (both logically valid) and the argument forms of denying the antecedent and affirming the consequent (both logically nonvalid), here called “the MP-quartet” for short. The modus ponens

From  {if A then B,A}  inferB

is the best known and most important inference rule in deductive logic. It is endorsed by practically all people (Rips, 1994). If the premises are uncertain and the conditional is interpreted as a conditional event we have in terms of point probability:

(8) $$\begin{array}{r} \text{From }\left\{P\left(B|A\right)=x,P\left(A\right)=y\right\}\;\text{infer}P\left(B\right)=z,\;\text{and }z\in \left[xy,1-y+xy\right]. \end{array}$$

For the lower and upper bounds for the three other rules see for example (Pfeifer and Kleiter, 2005).

**Figure 5:**
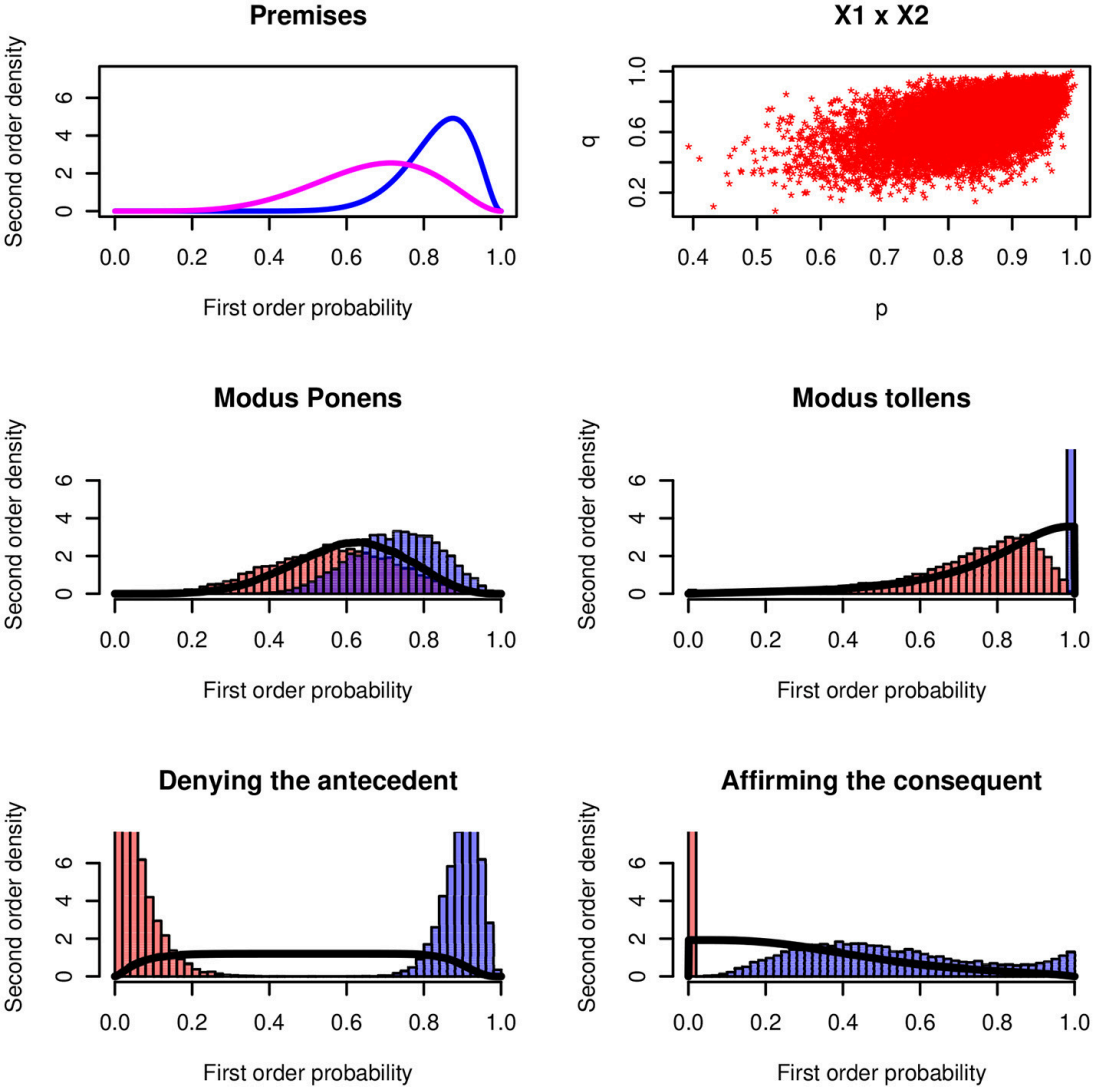
Four inferences rules. **(Upper panels)** Probability distribution of the minor premise and the major premises *P*(*B*|*A*). Histograms of the lower and upper probabilities of the four rules. The continuous distributions show the distributions of the probability of being coherent.

Figure 5 shows the results for the four inference rules for a numerical example. The premises have the distributions *X* ~ *Be*(15, 3), *Y* ~ *Be*(6, 3), and the Gaussian copula *ρ* = 0.5^4^.

The modus ponens has a maximum probability of being coherent that is close to the distribution of the minor premise *P*(*A*). For the modus tollens the maximum probability is at 1.0. The modus tollens is the strongest inference rule (Pfeifer and Kleiter, 2005, 2006b). Psychologically the modus tollens is difficult and complex; it's a “backwards” rule and it involves two negations. Usually the endorsement is much lower than for the modus ponens.

The two logically non-valid inference forms lead to probabilities of being coherence that are close to uniform distributions. In a psychological investigation the two rules should stick out by the *variance* of the probability judgments. More or less any probability judgment in [0, 1] is coherent.

The following section applies distributional imprecision to a series of examples. Most of them are well-known from the psychological literature but the inclusion of imprecision into their analysis leads to new properties and results.

## 3. Applications and examples

### 3.1. Natural sampling

One of the best known fallacies in judgment under uncertainty is the base rate neglect (Kahneman and Tversky, 1973; Bar-Hillel, 1980; Koehler, 1996). A doctor may, for example, neglect the prevalence of a disease and concentrate only on the likelihood of a symptom given the disease. While this is often a major fallacy, there are situations in which base rate neglect is completely rational. This holds also for beta distributions: Assume the shape parameters *α* and *β* of a distribution *Be*(*α*, *β*) are equal to the frequency of a binary feature in a sample of *n* observations, *n* = *α* + *β*. Split the total sample into two subsamples so that the sample sizes add-up to *n*. So the subsample sizes are not pre-planned. In statistics this is called *natural sampling* (Aitchison and Dunsmore, 1975). We have *Be*(*α*_1_, *β*_1_), *Be*(*α*_2_, *β*_2_) and *α* = *α*_1_ + *α*_2_ and *β* = *β*_1_ + *β*_2_, and *n* = *α*_1_ + *α*_2_ + *β*_1_ + *β*_2_. For natural sampling it was proven (Kleiter, 1994) that the base rates in Bayes' Theorem are “redundant” and may be ignored. The result for precise probabilities has often been used by Gigerenzer within his frequentistic approach (Gigerenzer and Hoffrage, 1995; Kleiter, 1996).

Ignoring base rates may not only be rational for precise but also for imprecise probabilities. For natural sampling it holds that if the knowledge about the prevalence of a disease *H* is represented by the beta *P*(*H*) ~ *Be*(*α*, *β*) and the conditional probabilities of a symptom *D* are represented by the betas *P*(*D*|*H*) ~ *Be*(*α*_1_, *β*_1_) and *P*(*D*|¬*H*) ~ *Be*(*a*_2_, *b*_2_), then the posterior distribution of the disease given the symptom *D* is simply

(9) $$\begin{array}{r} \begin{array}{c} P\left(H|D\right)\sim Be\left(\alpha_{1},\alpha_{2}\right),\\ \text{mean }=\frac{\alpha_{1}}{\alpha_{1}+\alpha_{2}},\;\text{variance }=\frac{\alpha_{1}\alpha_{2}}{{\left(\alpha_{1}+\alpha_{2}\right)}^{2}\left(\alpha_{1}+\alpha_{2}+1\right)}. \end{array} \end{array}$$

If frequencies are used to update subjective probabilities and if (and only if) natural sampling conditions hold, the resulting degrees of belief remain in the family of beta distributions, i.e., the distributions are natural-conjugates. Note that (relative) frequencies and probabilities are not the same. The frequencies are used to estimate probabilities and the representation of the imprecision of these estimates is an integral part of any statistical approach. The property of natural sampling extends to multivariate Dirichlet distributions and is thus helpful to represent imprecise degrees of belief in more complex environments. If the natural sampling assumption is dropped, then vines and copulas offer elegant methods to model the representation and propagation of degrees of belief.

### 3.2. Rips inference tasks

To show that a wide range of logical inference tasks can be modeled within the distributional approach we discuss very briefly two examples from Rips (1994). Rips compared the predictions of his proof-logical PSYCOP model with empirical data. He investigated 32 inference problems of classical sentential logic. Among them the following one:

IF Betty is in Little Rock THEN Ellen is in Hammond. Phoebe is in Tucson AND Sandra is in Memphis. Is the following conclusion true: IF Betty is in Little Rock THEN (Ellen is in Hammond AND Sandra is in Memphis) (Rips, 1994, p. 105).

When we represent the conditional by a conditional event^5^ and first introduce precise probabilities:

$$\begin{array}{l} P(B|A)=x\\ P(C\wedge D)=y\\ \bar{P(B\wedge D|A)\in [0,x]} \end{array}$$

The interval probability of the conclusion, *P*(*B*∧*D*|*A*)∈[0, *x*], is easily obtained after seeing that the probability of the conjunctive premise is irrelevant. *P*(*D*) is greater than *P*(*C*∧*D*) and may maximally be 1. The upper probability of the conclusion is thus $P\left(B\wedge D|A\right)=\frac{P\left(A\wedge B\wedge D\right)}{P\left(A\right)}$ and $P\left(B\wedge D|A\right)=P\left(D\right)\frac{P\left(A\wedge B\right)}{P\left(A\right)}=P\left(B|A\right)$. Analog relationships hold for the probability distributions.

In a second step beta distributions for the premises are introduced, say *X* and *Y*, and by stochastic simulation the distributions for the lower and upper probabilities and the distribution of the probability of being coherent are determined. The distribution of the probability of being coherent is practically uniform over the range between 0 and the mean of *X*. For high probabilities of the conditional premise the inference is inconclusive. In classical logic and in the proof-logical approach of Rips the inference is valid.

Here is a second example (Example M in Rips, 1994, p. 151):

$$\begin{array}{l} \neg A\\ B\neg \\ \bar{\left(A\wedge C)\wedge (B\vee D\right)} \end{array}$$

With *P*(¬*A*) = *x* and *P*(*B*) = *y* the probability of the conclusion is in the interval *z*∈[max{0, *x*+*y*−1}, 1}]. The lower probability is the same as the lower probability of a conjunction. If *x* and *y* are less than 0.5, then the inference is noninformative and the distribution of the probability of being coherent is a uniform distribution. The inference was endorsed by only 22.2% of the participants.

We next turn to an example from the judgment under uncertainty domain. It may be considered as an example of Ockham's razor (Tweney et al., 2010) where less is more.

### 3.3. The doherty task

For the conjunction of *n* events we have: If *P*(*D*_*i*_) = *α*_*i*_ for *i* = 1, …, *n*, then

(10) $$P(D_{1}\wedge D_{2}\wedge \ldots \wedge D_{n})\in \left[\max\left\{\sum_{i=1}^{n}\alpha_{i}-(n-1)\},\min\{\alpha_{i}\}\right\}\right].$$

This is a straightforward generalization of the elementary conjunction rule. Such generalizations were first investigated by Gilio (2012) and are also studied in Wallmann and Kleiter (2012a,b, 2014a,b). There is a psychologically interesting property of such generalizations. It is the phenomenon called *degradation*. As *n*, the number of events in the generalization, increases the inferences become less and less informative. More information leads to less conclusive inferences.

An example in the field of judgment under uncertainty is the so called pseudodiagnosticity task introduced by Michael Doherty (Doherty et al., 1979, 1996; Tweney et al., 2010; Kleiter, 2013). It was analyzed with second-order distribution by Kleiter (2015).

Assume you are a physician and you are 50% sure that one of your patients is suffering from disease *H*, *P*(*H*) = 0.5. You know that the probability that if the patient is suffering from *H*, the patient shows symptom *D*_1_ is 0.7, *P*(*D*_1_|*H*) = 0.7. You may obtain just one more piece of information. There are three options:

1. *P*(*D*_2_|*H*), the probability of a second symptom given the presence of the disease,
2. *P*(*D*_1_|¬*H*), the probability of the first symptom given the absence of the disease, or
3. *P*(*D*_2_|¬*H*), the probability of the second symptom given the absence of the disease.

What is your choice?

Most people select *P*(*D*_2_|*H*). Actually *P*(*D*_1_|¬*H*) is the best choice. With *P*(*D*_1_|¬*H*) Bayes' theorem gives the posterior probability

(11) $$\begin{array}{r} P\left(H|D_{1}\right)=\frac{P\left(H\right)P\left(D_{1}|H\right)}{P\left(H\right)P\left(D_{1}|H\right)+\left[1-P\left(H\right)\right]P\left(D_{1}|\neg H\right)}. \end{array}$$

Before any of the three options is selected, the posterior probability is in the interval (Tweney et al., 2010)

(12) $$\begin{array}{r} P\left(H|D_{1}\right)\in \left[\frac{P\left(H\right)P\left(D_{1}|H\right)}{P\left(H\right)P\left(D_{1}|H\right)+1-P\left(H\right)},\;1\right]. \end{array}$$

If however, as most participants do, *P*(*D*_2_|*H*) is selected, then the interval is

(13) $$\begin{array}{r} P\left(H|D_{1},D_{2}\right)\in \left[\frac{P\left(H\right)P\left(D_{1},D_{2}|H\right)}{P\left(H\right)P\left(D_{1},D_{2}|H\right)+1-P\left(H\right)},\;1\right]. \end{array}$$

The interval in (13) is wider than the interval in (12) as

$$P\left(D_{1},D_{2}|H\right)\leq \min\left\{P\left(D_{1}|H\right),P\left(D_{2}|H\right)\right\}\leq P\left(D_{1}|H\right).$$

Selecting *P*(*D*_1_|¬*H*) results in a precise point probability while selecting *P*(*D*_2_|*H*) results in an interval that is *wider* than the initial one.

**Figure 6:**
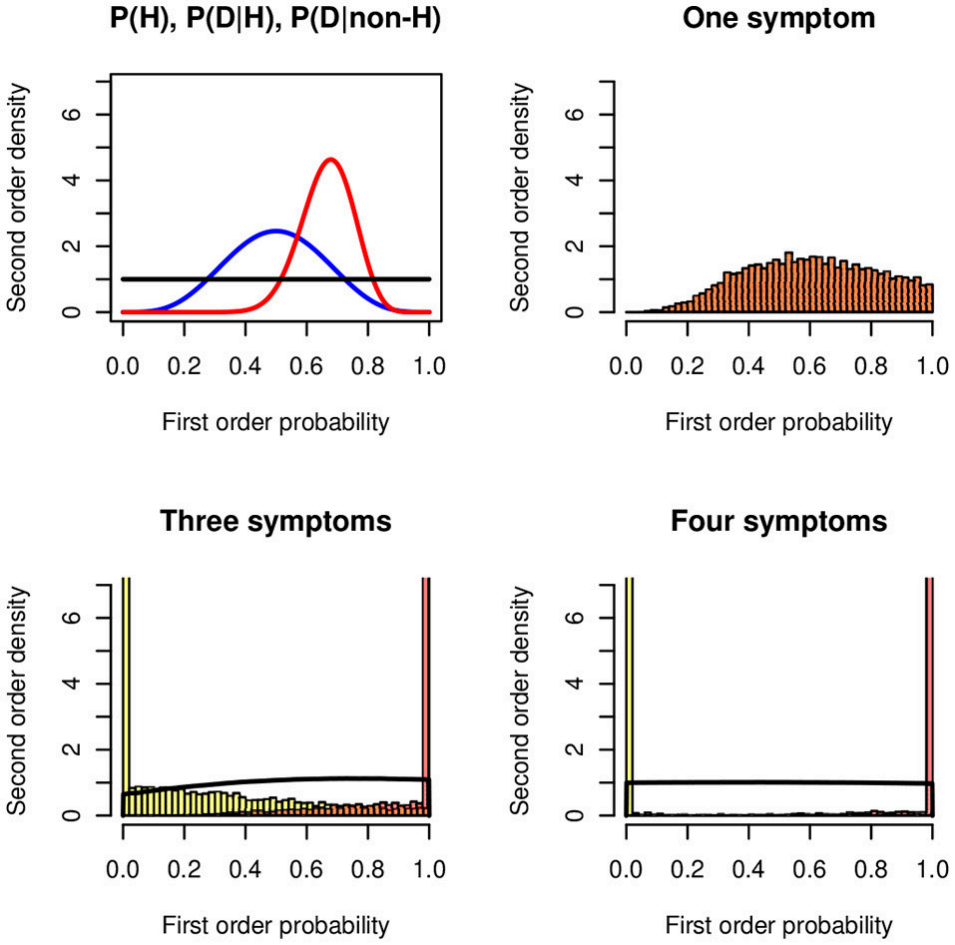
Degradation in the Doherty task. **(Top left panel)** The symmetric beta distributions *Be*(5, 5) of *P*(*H*) (blue), *Be*(20, 10) for *P*(*D*|*H*) (red), and the uniform distribution *Be*(1, 1) for *P*(*D*|¬*H*) (black). **(Top right panel)** Second-order posterior distribution of the probability of *H* when the distributions of the likelihoods *P*(*D*|*H*) and *P*(*D*|¬*H*) are known. **(Bottom panels)** Lower and upper distributions of the probability of *H* when the distributions of the likelihoods of three **(Left panel)** and four **(Right panel)** symptoms are known; all likelihood distributions are *Be*(20, 10) and *Be*(1, 1), respectively. The black line shows the probability of being coherent.

If we continue to select only the “affirmative ” likelihoods given *H* and not those given ¬*H*, then the intervals get wider and wider and after a few more steps become noninformative, that is, [0, 1]. The additional information imports noise. Figure 6 shows an example for *P*(*H*) ~ *Be*(5, 5), *P*(*D*_*i*_|*H*) ~ *Be*(20, 10), and *P*(*D*_*i*_|¬*H*) ~ *Be*(1, 1). For *i* = 1 there is one posterior distribution, the lower and the upper distributions coincide; for *i* = 3 and *i* = 4 the lower and upper distributions get close to 0 and 1. The probability of being coherent becomes a uniform distribution. One reason that contributes to the degradation effect are the unknown probabilities of the conjunctions *P*(*D*_1_|*H*)∧…∧*P*(*D*_*n*_|*H*) and *P*(*D*_1_|¬*H*)∧…∧*P*(*D*_*n*_|¬*H*).

The Doherty task demonstrates that we should compare the results from experimental groups with those from control groups. The preference for selecting the affirmative likelihood only is seen as a confirmation bias: people do not consider alternative hypotheses. The phenomenon that more information may induce more imprecision has been studied in Wallmann and Kleiter (2012a,b, 2014a,b) and Kleiter (2013).

Technically the analysis of a *multivariate* problem like the Doherty task requires stochastic simulation in *vines*. “Vines are graphical structures that represent joint probabilistic distributions. They were named for their close visual resemblance to grapes …” Kurowicka and Joe (2011, p. 1). Vines may be compared to Bayesian networks. In psychology Bayesian networks were used, for example, to model uncertain reasoning (Oaksford and Chater, 2007), to model causal reasoning (Tenenbaum et al., 2007), word learning (Xu and Tenenbaum, 2007), or to model cognitive development (Gopnik and Tenenbaum, 2007). Bayesian networks encode conditional independencies and represent the (usually precise) joint probabilities in tables. Vines encode marginal probabilities and (partial) correlations, or more generally, copulas. Psychologically it is more plausible that humans encode multivariate uncertain structures by their (conditional) dependencies and not by their (conditional) independencies. Moreover, encoding marginal probabilities is much easier than encoding multivariate probability tables. There is no space here for further speculations. For the mathematical treatment of vines the reader is referred to Kurowicka and Cooke (2004, 2006), Kurowicka and Joe (2011), and Mai and Scherer (2012).

A psychologically interesting difference between Bayesian networks and vines is that vines encode dependencies “directly” by (partial) correlations (actually copulas) and not by conditional probabilities. It is highly plausible (but seldom investigated) that humans encode the strength of a dependence not by a probability table but by a one-dimensional quantity.

While Bayesian networks rely on (conditional) independence assumptions, vines rely on *copulas*. Copulas encode dependencies. To keep the present text simple we use Gaussian copulas (correlations) only (see Equation 4). The recent advances in the theory of copulas and vines, and the development of software for the *simulation* methods allow to model multivariate imprecise inference. There is not enough space here to discuss a more complex example, but see the study of the Doherty's pseudodiagnosticity task in (Kleiter, 2015). The suppression task in the following section involves three variables.

### 3.4. Suppression task

The Suppression Task was introduced by Byrne (1989). She observed that while a simple modus ponens is endorsed by nearly all people, the endorsement decreases substantially when an *additional* conditional premise is introduced. The additional premise *suppresses* the acceptance of the conclusion. Table 4 shows Byrne's by now classical example:

**Table 4:** The various premises and the conclusion in the Suppression Task.

*P*1	Main conditional	If Mary has an essay to write, then she will study late in the library.
*P*2*a*	Additional conditional	If the library is open, then she will study late in the library.
*P*2*b*	Alternative conditional	If Mary has some textbook to read, then she will study late in the library.
*P*3	Categorical premise	Mary has an essay to write.
*C*	Conclusion	Mary will study late in the library.

The simple modus ponens “from {*P*1, *P*3} infer *C*” is endorsed by 96% of the participants in Byrne's Experiment 1. When the additional premise P2a is included, “from {*P*1, *P*2*a, P*3} infer *C*” the endorsement drops to 38%. When the alternative premise *P*2*b* is introduced, “from {*P*1, *P*2*b, P*3} infer *C*,” the endorsement is the same as for the simple modus ponens.

In an abstract formal system the second premise is logically and probabilistically irrelevant. It has no impact upon the conclusion, neither upon its truth nor upon its probability. Attending to the semantic content of the conditional premises, however, leads to a reinterpretation of the inferences. The conditionals *P*1 and *P*2 have the same consequent and Mary can only study late in the library if the library is open. Thus for the additional conditional the semantic content (Byrne, 1989) invites a conjunctive interpretation of the antecedent, {if *A*∧*B* then *C, A*}. The alternative conditional P2b, however, invites a disjunctive interpretation of the antecedent, {if *A* ∨ *B* then *C, A*}.

The distributional interpretation of the three different inferences are:

1. Simple modus ponens: *P*(*C*|*A*) = *X*, *P*(*A*) = *Y*.
2. Conjunctive antecedent: *P*(*C*|*A* ∧ *B*) = *X* , *P*(*A* ∧ *B*) = *Y*. We note that if *P*(*A*) = *x* and *P*(*B*) is unknown and thus may have any value between 0 and 1, *P*(*A* ∧ *B*) is in the interval [0, *x*]. The bounds for the modus ponens are *z*∈[0, 1−*x*+*xy*]
3. Disjunctive antecedent: *P*(*C*|*A* ∨ *B*) = *X* , *P*(*A* ∨ *B*) = *Y*. *P*(*B*) is unknown and *P*(*A* ∨ *B*) may have any value in the interval [*x*, 1]. The bounds for the modus poens are *z*∈[*xy, y*].

**Figure 7:**
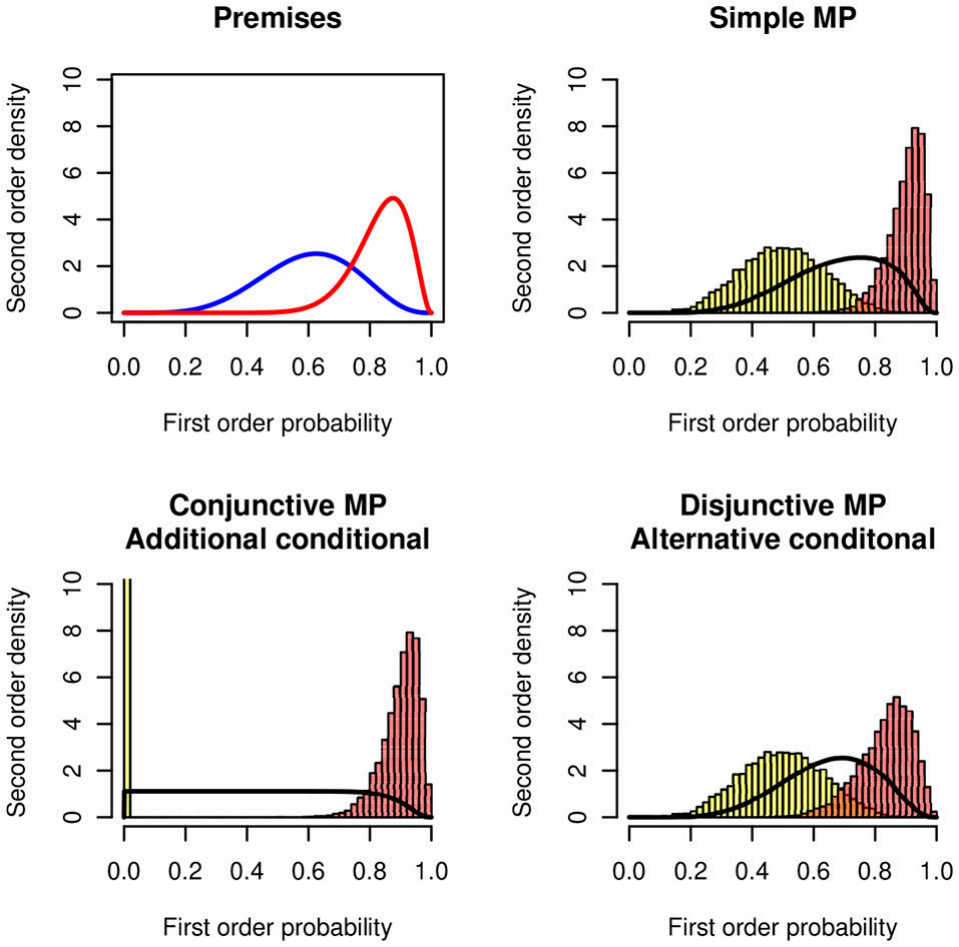
Modus ponens in the Suppression Task. **(Top panels)** Probability distributions of the premises *P*(*C*|*A*) ~ *Be*(15, 3) **(Right)** and *P*(*A*) ~ *Be*(6, 4) **(Left)**. Simple modus ponens: Lower and upper histograms of the probability of the conclusion *P*(*C*). **(Bottom panels, Left)** The premises are interpreted as a conjunction, *P*(*C*|*A* ∧ *B*) and *P*(*A* ∧ *B*). **(Right)** The premises are interpreted as a disjunction , *P*(*C*|*A* ∨ *B*) and *P*(*A* ∨ *B*).

Figure 7 shows the distributions of the lower and the upper bounds and of the probability of being coherent. The example uses the following distributions: (1) For the simple modus ponens *P*(*A*) = *X* ~ *Be*(10, 5) and *P*(*C*|*A*) = *Y* ~ *Be*(20, 5). (2) For the conjunctive interpretation (additional conditional) *P*(*A* ∧ *B*) = *X* ~ *Be*(10, 5) and *P*(*C*|*A* ∧ *B*) = *Y* ~ *Be*(20, 5) (3) For the disjunctive interpretation (alternative conditional) *P*(*A* ∨ *B*) = *X* ~ *Be*(10, 5) and *P*(*C*|*A* ∨ *B*) = *Y* ~ *Be*(20, 5).

In the figure the simple modus ponens and the disjunctive antecedent (If Mary has an essay to write or if Mary has a textbook to read) lead to very similar results. The conjunctive antecedent (If Mary has an essay to write and if the library is open) leads to a very flat distribution. The distribution of the lower bound is degenerate at zero. The probability of the conjunction is much lower than the probability of the disjunction.

The distributional approach models the results of the Suppression Task pretty well. Moreover, it provides quantitative predictions for the differences in the various experimental conditions.

The suppositional interpretation of an “if H then E” sentence assumes *H* to be true. Also in a conditional probability *P*(*E*|*H*) the event *H* is assumed to be true. Jeffrey pointed at cases where observations are blurred. Under candle light the color of an object may be ambiguous. How to condition on soft evidence? Jeffrey was the pioneer of the analysis of soft evidence to which we will turn next.

### 3.5. Soft evidence

Usually *conditioning* updates probabilities in the light of *hard* evidence, that is, the conditioning event is supposed to be *true*. But what if the conditioning event is only uncertain? Jeffrey introduced “Jeffrey's rule,” a proposal of how to update probabilities by *soft* evidence (Jeffrey, 1965, 1992, 2004). Historically the problem was ready posed by Donkin (1851) and his solution is equivalent to Jeffrey's rule (for a proof see Draheim, 2017). Draheim gives an overview of the literature in Appendix A of his monograph. Jeffrey's rule has been criticized by several authors (Levi, 1967; Diaconis and Zabell, 1982; Wedlin, 1996; Halpern, 2003; Jaynes, 2003). The rule is *non-commutative*, i.e., it is not invariant with respect to the order of updating. Moreover, it involves an independence assumption. For a psychological investigation of Jeffrey's rule see Hadjichristidis et al. (2014).

In the present approach it is straightforward to update probabilities by evidence that is probable only. We have two random variables *X* and *Y* (first-order probabilities). We want to know the (second-order) distribution of *Y* given a fixed value of *X*. The problem is analog to a regression problem in statistics: The distribution of *Y* is predicted on the basis of a given value of *X* = *x* . The distributional approach offers a direct representation of Jeffrey's problem.

**Figure 8:**
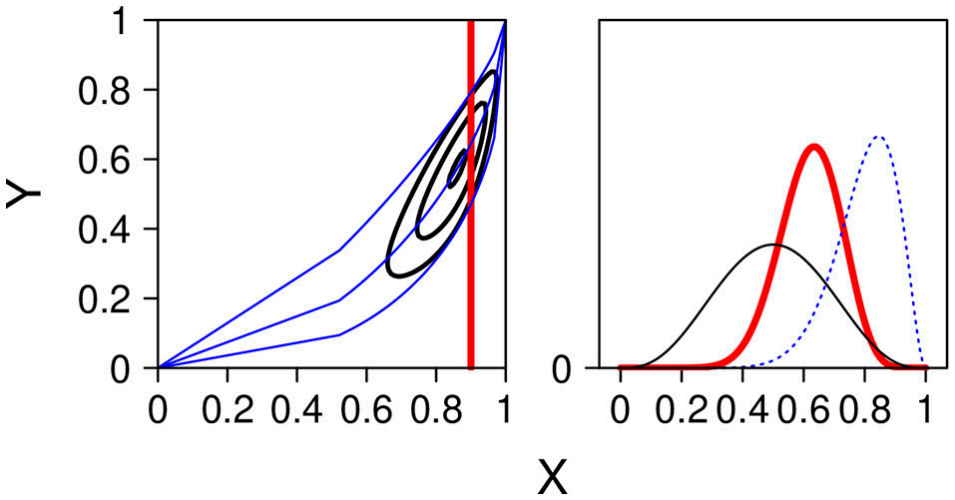
**(Left panel)** Contour lines of the joint distribution with the marginals *X* ~ *Be*(9, 3) and *Y* ~ *Be*(4, 4), and Spearman correlation τ = 0.5. Regression line at *x* =.9 (quantile at 0.5) together with 90 % confidence band (quantile at 0.05 and 0.95). **(Right panel)** The two marginal betas and the conditional distribution *p*(*y*|*x*_0_ = 0.9) along the vertical line in the contour plot.

Figure 8 shows a numerical example. On the left side the unit square [0, 1]^2^ and the contour lines from the bivariate joint distribution resulting from two beta marginals and a Spearman copula^6^. On the right side the two marginals and the distribution of *Y* at *X* = 0.9. The contour lines and the distribution at the cutting point 0.9 is obtained by stochastic simulation.

## 4. Discussion

We have distinguished logical, probabilistic, and statistical principles and argued that for a plausible model of human reasoning ingredients from all the three domains are necessary. We have seen that the constraints of probability logic induce only lower and upper probabilities, or lower and upper distributions in the case of imprecision; they do not lead to exact point probabilities, or to just one distribution in the case of imprecision. To overcome this kind of indeterminacy we have introduced the concept of the *probability of being coherent*. One may follow the proposal of Smets (1990) and distinguish *credal* and *pignistic* degrees of belief, corresponding to the whole distribution for the cognitive representation and the maximum for selecting just one favorite value. It is rational to base one's decisions on values obtaining a maximum probability of being coherent.

We have investigated the differences between the logical conjunction and the conditional. For not too extreme probabilities these differences may be small, so small that it will be impossible to distinguish the two interpretations empirically. We observed that in typical truth table tasks about twenty percent of the participants interpret if-then sentences as conjunctions (Fugard et al., 2011; Kleiter et al., 2018). In the context of everyday conversation, say, the different interpretations would not matter. We compared the sensitivity of the differences between the logical operators by the Kullback-Leibler distances between their distributions. The distance of an inferred distribution, inferred from a logical argument, from the uniform distributions, as a standard of ignorance, is an indicator of the informativeness and strength of the argument.

We remembered that neglecting base rates may be rational under natural sampling conditions. This property holds for beta distributions, their expected values and variances. We have demonstrated how typical tasks of deductive reasoning (Rips, 1994) can be cast into a probabilistic format including imprecision. A paradoxical property is observed in Doherty's information seeking task (Doherty et al., 1979; Tweney et al., 2010; Kleiter, 2015): Sampling more and more information from just one experimental condition, without sampling from a control condition, leads to less and less precise conclusions. The suppression task (Byrne, 1989) was among the first tasks framed and analyzed in a probabilistic format (Stevenson and Over, 1995). Expressing the implicit assumptions by second order probability distribution predicts the empirical results reported in the literature. Jeffrey's proposal of how to update probabilities by uncertain evidence is well known as Jeffrey's rule (Jeffrey, 1965). In a bivariate model with two first order probabilities *X* and *Y* treated as random variables the problem becomes a typical regression problem, predicting the distribution of *Y* given a value of *X*.

Gigerenzer et al. (1991) proposed a probabilistic mental model (PMM) of confidence judgments. The model was introduced and demonstrated by the experimental paradigm of *city size judgments*. In the first of two experiments twenty five German cities with more than 100,000 inhabitants were selected. Participants were presented all 300 pairs of the cities and asked to decide which one has more inhabitants. In addition, the participants rated how sure they were that each of their choices was correct.

Using just one quantitative property, city size, underlying all questions in the experimental procedure introduced a big difference with respect to the general knowledge almanac questions widely used in other studies of overconfidence^7^.

The data may be looked at from the perspective of the method of paired comparison (Thurstone, 1927). Processing the data with Thurstone's probabilistic model of paired comparison one would introduce a normal distribution for the size of each of the cities. Such a probability distribution models the participant's knowledge about the size of a city and the precision of this knowledge. The confidence judgment then becomes a function of the differences in the location and spread of these distributions. The distributions are thus not second order probability distributions, but distributions over a quantitative property, here the number of inhabitants of a city. The property is imprecise (compare the intervals in Figure 2 of Gigerenzer et al., 1991), not the probability^8^. The same holds for the cues in the PMMs.

I consider the analyses presented in this contribution as part of a thorough task analysis of reasoning tasks. Task analysis is a prerequisite for a good psychological investigation. The results of our analyses show how difficult it may be to run a good reasoning experiment. A major problem, e.g., is how to manipulate and measure imprecision. Another problem is that inferences with the same logical operators or the same logical inference rules may be different for different levels of the probabilities of the premises. High probabilities may lead to one result, low probabilities to a different one. Results may also not be invariant with respect to positive or negative correlations of the involved uncertain quantities and risks.

Modeling imprecise judgments has a long history. It started with Gauss and his analysis of human judgment errors in astronomical observations. It continued in the nineteenth century with Weber's and Fechner's just noticeable differences, thresholds and psychophysical functions. The probabilistic modeling of sensory data by von Helmholtz pioneered present day's Free Energy Principle. Thorndike introduced the law of comparative judgment. In the second half of the twentieth century signal detection theory, stimulus sampling theory, stochastic choice theory, Brunswick's lens model, stochastic response models, neural networks, and decision theory took up the problem. At the beginning of the twenty first century computational neuroscience contributed substantially to model imprecision in information processing.

Models of the functioning of the brain claim that the neuronal processes underlying cognitive processes like memory, perception, or decision making are inherently *stochastic* and *noisy*. A good example is the work of Rolls and Deco (2010). Spike trains of neurons follow Poisson distributions, cell assemblies are modeled by mean-field analysis and the dynamics of elementary decision processes are simulated by integrate-and-fire neural networks. The authors observe that “…if a decision must be made based on one's confidence about a decision just made, a second decision-making network can read the information encoded in the firing rates of the first decision-making network to make a decision based on confidence …” (Rolls and Deco, 2010, p. 167). A probability assessment is a read-out of one's own confidence, the product of an auto-epistemic self-monitoring process (Rolls and Deco, 2010, p.196ff.). The assessment might correspond to the point of maximum probability of being coherent.

Precision plays an important role in the theories of free energy, active inference, and predictive coding (Friston, 2010; Buckley et al., 2017). In a task in which the participants had to decide on the direction of a set of systematically moving dots in a set of randomly moving dots the precision of the responses was related to the response times. It was shown that the precision of the responses was controlled (among other locations) in the posterior parietal cortex (FitzGerald et al., 2015). Precision may be modulated by neurotransmitters. Friston et al. (2012), for example, hypothesized that precision is related to dopamin.

In probability logic all operators and inference rules infer interval probabilities. Using conclusions iteratively would require to propagate lower and upper probabilities again and again. For a human brain to keeping track of lower and upper bounds will soon become too messy. One way out of the exploding complexity is to simplify and process the probability distributions of being coherent. To use a metaphor: In a cell assembly the distributions may result from the many single cell activations constrained by the coherence criterion.

## Author contributions

The author confirms being the sole contributor of this work and has approved it for publication.

### Conflict of interest statement

The author declares that the research was conducted in the absence of any commercial or financial relationships that could be construed as a potential conflict of interest.

## Footnotes

^1^For Adam's probabilistic validity in the more recent research on human reasoning see Kleiter (2018).

^2^From Equation (6) is may be seen that as *x* approaches 1 the bounds of the conditional approach the bounds of the conjunction in Equation (3).

^3^If the premises are specified by interval probabilities the situation gets more complicated and requires the concepts of g-coherence (Gilio, 1995) or the avoidance of sure loss (Walley, 1991). We do not need the concepts here.

^4^Denying the antecedent and affirming the consequent degenerate at 0; the modus tollens degenerates at 1.

^5^Note that Rips (1994, p. 125) prefers the suppositional interpretation of the conditional; the domain of a conditional consists only of those possibilities in which the antecedent is true. PSYCOP rejects the paradoxes of the material implication!

^6^In the literature Spearman correlation copulas are often preferred to Gaussian copulas as they keep the distribution of the marginals and the correlation independent.

^7^The study of overconfidence can be tricky as overconfidence for *E* goes hand in hand with underconfidence for non-*E*. Scoring rules avoid this problem (Kleiter et al., 2002).

^8^It may be mentioned that the evaluation of the data by the method of paired comparison would allow to calculate several interesting statistics like item characteristics, the consistency of the judgments, or interindividual differences.
