# Supplementary material for: Imprecise Uncertain Reasoning: A Distributional Approach
Source: Front Psychol. 2018 Oct 26;9:2051. doi: 10.3389/fpsyg.2018.02051 (PMC6212601; doi:10.3389/fpsyg.2018.02051)
Supplement: Supplementary file 1 [file Data_Sheet_1.PDF]

```
# inf_quartet.R
# Investigating second order probability distributions for
# modus ponens, modus tollens, denying the antecedent, and affirming the consequent
rm(list = ls())  # clear all
# invoke the VineCopula package and the Cairo package
# Be sure that the packages and your R do have the same version numbers
require(VineCopula, quiet=TRUE, warn.conflicts = FALSE)
require(Cairo, quiet=TRUE, warn.conflicts = FALSE)
# ------------------------------------------------------------------------------
# Initialize
# ------------------------------------------------------------------------------
N = 50000        # number of simulations, reduce on a slow machine
nn = 999         # number of points on the X axis, streching the [0,1] interval
n = 2            # number of premises
a1 = 15; b1 = 3  # parameters of the beta distribution, categorical premise
a2 = 6; b2 = 3   # parameters of the beta distribution, conditional premise
type = 1         # type of copula, 1 selects the Pearson correlation
r = .5           # value of the correlation (first copula parameter)
#set.seed(527459) # introduce a seed
# ------------------------------------------------------------------------------
# Prepare arrays
# ------------------------------------------------------------------------------
w1 = rep(0, N)  # lower probabilities for MP
w2 = rep(0, N)  #                         MT
w3 = rep(0, N)  #                         DA
w4 = rep(0, N)  #                         AC

m1 = rep(0, N)  # upper probabilities for MP
m2 = rep(0, N)  #                         MT
m3= rep(0, N)   #                         DA
m4 = rep(0,N)   #                         AC

coherent1 = rep(0, (nn + 1)) # probabilities of being coherent, MP
coherent2 = rep(0, (nn + 1)) #                                  MT
coherent3 = rep(0, (nn + 1)) #                                  DA
coherent4 = rep(0, (nn + 1)) #                                  AC
step = 1 / nn                # step size on the X-axis, for histograms
px = seq(0, 1, step)
# The probability densities for the two premises ------------------------------
beta1 = dbeta(px, a1, b1)  # categorical premise
beta2 = dbeta(px, a2, b2)  # conditional premise
# ------------------------------------------------------------------------------
# Functions for the lower bounds of the MP, MT, DA, and AC
# The functions may easily be changed for other inference rules
# ------------------------------------------------------------------------------
W1 = function(x, y){ # MP
    x * y
}
W2 = function(x, y){ # MT, note the pairwise maximum
    pmax((1 - x - y) / (1 - y), (y + x - 1) / y)
}
W3 = function(x, y){ # DA
    (1 - y) * (1 - x)
}
W4 = function(x, y) { # AC
    rep(0, N) # the lower probability is 0, thus the vector of 0s
}
# ------------------------------------------------------------------------------
# Function for the upper bounds
# ------------------------------------------------------------------------------
M1 = function(x, y){
    1 - x + x * y
}
M2 = function(x, y){ # the upper probability is 1, thus the vector of 1s
```

```
    rep(1, N)
}
M3 = function(x, y){
    1 - y * (1 - x)
}
M4 = function(x, y) { # note the pairwise minimum
    pmin(x / y, (1 - x)/(1 - y))
}
# ------------------------------------------------------------------------
# Reorder the R-matrix from the standard order in math-books
#  (rows first, columns second)to R's column-major oder
# ------------------------------------------------------------------------
reorder = function(Vector, n) {
  Matrix = matrix(0, n, n)
  k = n * n
  for (i in 1:n) {
    for (j in 1:n) {
      Matrix[i,j] = Vector[k]
      k = k - 1
    }
  }
return(Matrix)
}
# ------------------------------------------------------------------------------
# Define the R-vine matrix, see e.g. Mai & Scherer (2012), p. 196 ff.
# See the documentation of the VineCopula packages (Schepsmeier et al.)
# ------------------------------------------------------------------------------
Vector = c(1, 1,
           0, 2)
Matrix = reorder(Vector, n)
# define R-vine pair-copula family matrix
Vector = c(0, type, # type is defined in the initialization section
           0, 0)
family = reorder(Vector, n)
# define R-vine pair-copula parameter matrix
Vector = c(0,  r,   # r is fixed in the initialization section
           0,  0)
par = reorder(Vector, n)
# define second R-vine pair-copula parameter matrix
Vector = c(0,  0, # no copulas with two parameters are used
           0,  0)
par2 = reorder(Vector, n)
# ---------------------------------------------------------------
# Define RVineMatrix object
# ---------------------------------------------------------------
RVM = RVineMatrix(Matrix=Matrix,family=family,par=par,par2=par2,names=c("V1","V2"))
# The following plotting is optional
# See the documentation of  the VineCopula package (Schepsmeier et al.)
# plot all trees with pair-copula families and
# theoretical Kendall s tau values as edge labels
#P = RVineTreePlot(data=NULL,RVM=RVM,tree="ALL",edge.labels=c("family","theotau"),P=NULL)
# manipulate the first matrix of x-y-coordinates
#P[[1]][1,] = P[[1]][1,]*2
# plot only the first tree with new coordinates
#RVineTreePlot(data = NULL, RVM = RVM, tree = 1, edge.labels = FALSE, P = P)
# -----------------------------------------------------------
# Simulate
# -----------------------------------------------------------
   simdata = RVineSim(N, RVM)
   p = qbeta(simdata[,1], a1, b1) # Get the inverse (quantiles) for the betas
   q = qbeta(simdata[,2], a2, b2)
# Get the lower and upper probabilities with the help of the functions W1, ...

   w1 = W1(p, q)
   w2 = W2(p, q)
```

```
    w3 = W3(p, q)
    w4 = W4(p, q)

    m1 = M1(p, q)
    m2 = M2(p, q)
    m3 = M3(p, q)
    m4 = M4(p, q)

 # All values between w and m are coherent, add 1, that is,
 # count the coherent cases between the lower and the upper probabilities
 for (i in 1:nn) {
   for (j in 1:N) {
      if (w1[j] < px[i] & px[i] < m1[j]) {
          coherent1[i]=coherent1[i] + 1
      }
      if (w2[j] < px[i] & px[i] < m2[j]) {
          coherent2[i] = coherent2[i] + 1
      }
      if (w3[j] < px[i] & px[i] < m3[j]) {
          coherent3[i] = coherent3[i] + 1
      }
      if(w4[j] < px[i] & px[i] < m4[j]) {
          coherent4[i] = coherent4[i] + 1
      }
    }
 }
 # Standardize the counts
    coherent1 = nn * coherent1 / sum(coherent1)
    coherent2 = nn * coherent2 / sum(coherent2)
    coherent3 = nn * coherent3 / sum(coherent3)
    coherent4 = nn * coherent4 / sum(coherent4)
 # ---------------------------------------------------------------------------------
 # Either plot on screen or write to postscript file
 # ---------------------------------------------------------------------------------
 # CairoPS(file="quartet.ps", width=6, height=6, bg="transparent")
 layout(matrix(c(1, 2, 3, 4, 5, 6), 3, 2, byrow = TRUE),
        width = c(4, 4, 4, 4, 4, 4), height = c(3, 3, 3, 3, 3, 3))
 ymax = 1.5 * max(max(beta1), max(beta2)) # maximum ordinate for the plots
 lw2 = 2    # define a line width
 lw3 = 2    # change to 3 is you like
 by = 0.02  # step size for histograms
 alpha = .5 # Tranparity for overlying colors
 # The two beta distributions of the premises ---------------------------------
 plot(px, beta1, type = "l", lwd = lw2, lty = 1, col = "blue",
      xlim = c(0, 1), ylim = c(0, ymax), xlab = "First order probability",
      ylab = "Second order density", main = "Premises", sub = "")
 lines(px, beta2, lwd = lw2, col = "red")
 # Scatter plot ---------------------------------------------------------------
 plot(p, q, col = "red", type = "p", pch = "*", main = "X1 x X2")
 # Modus ponens ---------------------------------------------------------------
 hist(w1, probability = TRUE,
      col = rgb(red = 1,green = 1,blue = 0, alpha = alpha),
      main = "Modus Ponens", xlim = c(0, 1), ylim = c(0, ymax),
      xlab = "First order probability",ylab = "Second order density",
      breaks = seq(0, 1, by = by))
 hist(m1, probability = TRUE,
      col = rgb(1, 0, 0, alpha = alpha), main = "", xlim = c(0, 1),
      ylim = c(0, ymax), breaks = seq(0, 1, by = by), add = TRUE)
 lines(px, coherent1, lwd = lw3, col = "black")
 # Modus tollens --------------------------------------------------------------
 hist(w2, probability = TRUE,
      col = rgb(1, 1, 0, alpha = alpha), main="Modus tollens", xlim = c(0, 1),
      ylim = c(0, ymax), xlab = "First order probability",
      ylab = "Second order density", breaks = seq(0, 1, by = by))
 hist(m2, probability = TRUE, col = rgb(1, 0, 0, alpha = alpha),
```

```
     main = "", xlim = c(0, 1), ylim = c(0, ymax), breaks = seq(0, 1, by = by),
     add = TRUE)
lines(px, coherent2, lwd = lw3, col = "black")
# Denying the antecedent --------------------------------------------------------
hist(w3, probability = TRUE,
     col = rgb(1, 1, 0, alpha = alpha),
     main = "Denying the antecedent", xlim = c(0, 1),
     ylim = c(0, ymax), xlab = "First order probability",
     ylab = "Second order density", breaks = seq(0, 1, by = by))
hist(m3, probability = TRUE,
     col = rgb(1, 0, 0, alpha = alpha),  main ="", xlim = c(0, 1),
     ylim = c(0, ymax), breaks=seq(0, 1, by = by), add = TRUE)
lines(px, coherent3, lwd = lw3, col = "black")
# Affirming the consequent ------------------------------------------------------
hist(w4, probability = TRUE, col = rgb(1, 1, 0, alpha = alpha),
     main = "Affirming the consequent", xlim = c(0, 1), ylim = c(0, ymax),
     xlab = "First order probability", ylab = "Second order density",
     breaks =seq (0, 1, by = by))
hist(m4, probability = TRUE, col = rgb(1, 0, 0, alpha = alpha),
     main = "", xlim = c(0, 1), ylim = c(0, ymax),
     breaks = seq(0, 1, by = by), add = TRUE)
lines(px, coherent4, lwd = lw3, col = "black")
#dev.off()
# -------------------------------------------------------------------------------
# Some statistics and approximations
# -------------------------------------------------------------------------------
# Means and sd of the distributions of the premises
mx = a1 / (a1 + b1)
my = a2 / (a2 + b2)
cat("\n Means of the categorical and conditional premises ", mx, my)
# Distributions of the lower and upper probabilities
cat("Means and standard deviations of the lower and upper probabilities")
m1.low = mean(w1)
m1.up = mean(m1)
sd1.low = sd(w1)
sd1.up = sd(m1)
cat("\n\n MP: Means low and up   ", m1.low, m1.up, " sds ", sd1.low, sd1.up)

m2.low = mean(w2)
m2.up = mean(m2)
sd2.low = sd(w2)
sd2.up = sd(m2)
cat("\n MT: Means low and up   ", m2.low, m2.up, " sds ", sd2.low, sd2.up)

m3.low = mean(w3)
m3.up = mean(m3)
sd3.low = sd(w3)
sd3.up = sd(m3)
cat("\n DA: Means low and up   ", m3.low, m3.up, " sds ", sd3.low, sd3.up)

m4.low = mean(w4)
m4.up = mean(m4)
sd4.low = sd(w4)
sd4.up = sd(m4)
cat("\n AC: Means low and up   ", m4.low, m4.up, " sds ", sd4.low, sd4.up)
# -------------------------------------------------------------------------------
cat("\n\nProbability distributions of being coherent")
approxi.low = W1(mx, my)
approxi.up  = M1(mx, my)
cat("\n MP: (Very) rough approximation low and up ", approxi.low, approxi.up)
approxi.low = W2(mx, my)
approxi.up  = 1
cat("\n MT: (Very) rough approximation low and up   ", approxi.low, approxi.up)
approxi.low = W3(mx, my)
approxi.up  = M3(mx, my)
```

```
cat("\n DA: (Very) rough approximation low and up   ", approxi.low, approxi.up)
approxi.low = 0
approxi.up  = M4(mx, my)
cat("\n AC: (Very) rough approximation low and up   ", approxi.low, approxi.up)
 m1 = which.max(coherent1)/nn
 m2 = which.max(coherent2)/nn
 m3 = which.max(coherent3)/nn
 m4 = which.max(coherent4)/nn
 cat("\nThe modes of the four distributions are ", m1, "  ", m2, "  ", m3, "  ", m4)
 cat("\n")
```
